# Mapping behavioral specifications to model parameters in synthetic biology

**DOI:** 10.1186/1471-2105-14-S10-S9

**Published:** 2013-08-12

**Authors:** Heinz Koeppl, Marc Hafner, James Lu

**Affiliations:** 1 ETH Zurich, Zurich, Switzerland; 2 IBM Zurich Research Laboratory, Rueschlikon, Switzerland; 3 Harvard Medical School, Boston MA, USA; 4 F. Hoffmann-La Roche, Basel, Switzerland

## Abstract

With recent improvements of protocols for the assembly of transcriptional parts, synthetic biological devices can now more reliably be assembled according to a given design. The standardization of parts open up the way for *in silico *design tools that improve the construct and optimize devices with respect to given formal design specifications. The simplest such optimization is the selection of kinetic parameters and protein abundances such that the specified design constraints are robustly satisfied. In this work we address the problem of determining parameter values that fulfill specifications expressed in terms of a functional on the trajectories of a dynamical model. We solve this inverse problem by linearizing the forward operator that maps parameter sets to specifications, and then inverting it locally. This approach has two advantages over brute-force random sampling. First, the linearization approach allows us to map back intervals instead of points and second, every obtained value in the parameter region is satisfying the specifications by construction. The method is general and can hence be incorporated in a pipeline for the rational forward design of arbitrary devices in synthetic biology.

## Introduction

Synthetic biology places emphasis on small, standardized molecular parts and devices, mostly operating at the transcriptional level [1,2]. With standardization comes the need for rigorous quantitative characterization of such devices and for a compositional theory to reliably build larger systems from small canonical circuits. For now most synthetic circuits implemented *in vivo *were constructed from a small number of components with topology and parameter values found by trial-and-error. The development of larger synthetic systems necessitates the use of appropriate design methodologies. *In silico *analyses can provide significant insights into the construction of complex synthetic systems, but due to the poor quantification of experimental and micro-environmental conditions, the predictive capability of *in silico *models for *in vivo *implementations remains limited. Apart from experimental limitations, modeling attempts to date most often make simplifying assumptions about all the perturbations that a synthetic construct is facing in vivo. For instance, only a few studies account for the large extrinsic noise [3-5] and in particular the one introduced by variations of plasmid copy number [6]. Incorporating those realistic *in vivo *constraints will make computational models more predictive, eventually enabling the upfront *in silico *optimization of transcriptional circuits. A first step toward this goal is to investigate the parameter dependency of certain behaviorial properties of a circuits. In systems biology attempts have already been made to address this problem, however, they either rely on purely local measures [7,8] such as considered in classical sensitivity analysis [9,10], or perform random parameter sampling [11] to determined parameter dependencies.

For a given circuit topology, kinetic parameters and other parameters that are involved in controlling the expression level of molecular species (e.g. promoter activity or number of ribosome binding sites) are important design parameters in synthetic biology. A major challenge is to find a set of parameters that satisfies the behavioral specification of a device [12]. Computer science offers various languages to formally define the proper functioning of a piece of code or hardware. Such specification languages of formal verification are used to check important behavioral properties, such as liveness, safety or fairness [13]. One convenient way to specify such properties is to use *temporal logic*, which is considered an extension of classical propositional reasoning, where propositional variables may change their truth values over time. A prominent such logic is the linear temporal logic (LTL), where the truth value of the propositions is interpreted over a linear timeline [13]. Such techniques were already applied to investigate robustness of computational models in system biology [14].

**Figure 1:**
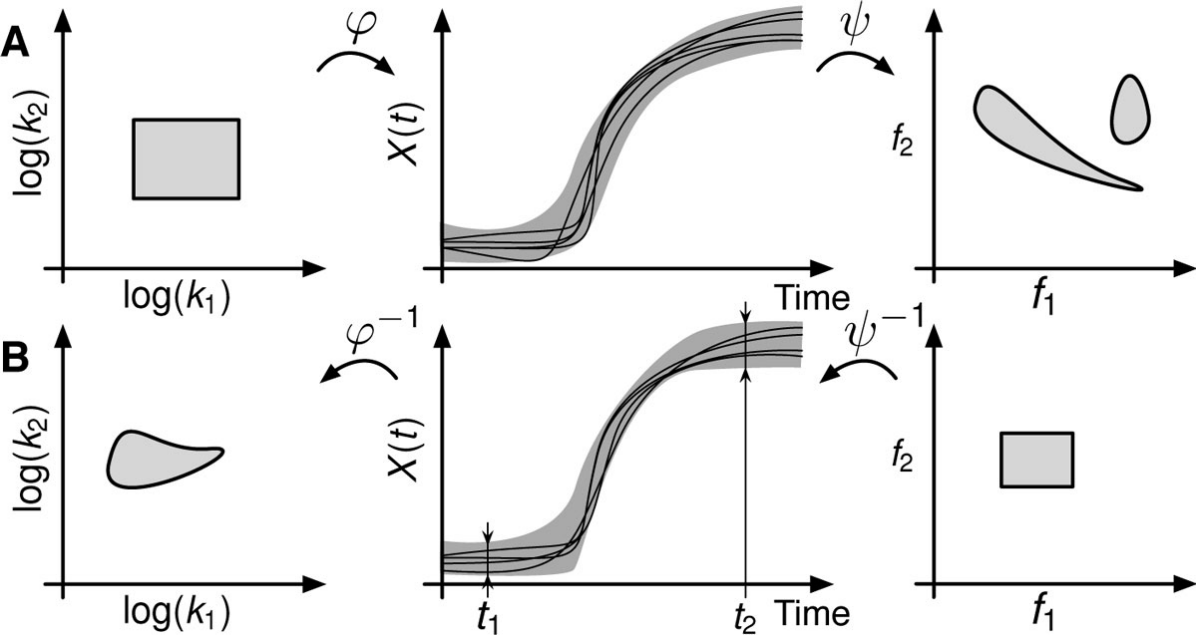
(A) The forward problem of defining a parameter set from which trajectories and their behavioral features are computed. (B) The inverse problem of finding a parameter regions for a predetermined behavioral specification region *S*. Columns from left to right correspond to parameter space, trajectory space and behavioral feature space, respectively. Connected convex sets can map to nonconvex non-connected regions.

Mathematically, the design problem is an inverse problem and hence inherits the general feature of such problems, namely ill-posedness [15,16]. More specifically, for a certain behavioral specification one aims to find the corresponding parameter set that gives rise to such behavior. An simple example for a quantity in feature space could be the concentration of a molecular species at particular time-points. The problem is closely related to parameter optimization and even more so to robust optimization, where an objective function - generally encoding some behavioral constraint (e.g. making model trajectories close to the measurements) - is optimized to yield the optimal parameter set. Ill-posedness refers to the observation that two close-by points in specification or behavioral feature space may map to very distant points in the parameter space, indicating that this mapping is generally not contractive but rather expansive. The inverse and corresponding forward problem is illustated in Figure 1.

In the current analysis we restrict ourselves to models obeying the reaction rate equation and hence constitute a set of nonlinear ordinary differential equations. In general, connected domains may map to disconnected domains, for instance if the dynamical system contains bifurcation points (e.g. see Figure 1). For the proposed linearization approach we will further restrict ourselves to connected domains in the respective image space. Moreover, we will not resort to specifying behavior through temporal logics but will define general *specification functionals*. These are mappings *ψ *from an appropriate function space *χ *of *n*-dimensional trajectories (e.g. $L_{2}(\left[0,T\right],{\mathbb{R}}^{n}$)) to the *m*-dimensional reals and we choose the form

$$\psi \left(x\right)\equiv \int_{0}^{T}g\left(s,x\left(s\right)\right)\text{d}s$$

with x∈X and the feature kernel $g:{\mathbb{R}}_{\geq 0}\times {\mathbb{R}}_{\geq 0}^{n}\to F$, where $F\subseteq {\mathbb{R}}^{m}$. A special and more tractable version of the kernel is the convolution, i.e. *g*(*t*, *x*(*t*)) = *h*(*T − t*)*x*(*t*). In the following we will only require the map *x *→ *g*(*·*, *x*) to be once-differentiable. With this, we can define the forward map from a *p*-dimensional parameter space to the feature space as the composition *F ≡ ψ *ο *φ*, with $\varphi :{\mathbb{R}}^{p}\to X$. The trajectories x∈X are generated by the reaction rate equation

(1) $$\frac{\text{d}}{\text{d}t}x\left(t\right)=Nv\left(x\left(t\right),k\right)\;\text{and}\;x\left(0\right)=x_{0}\in {\mathbb{R}}_{\geq 0}^{n},$$

with the stoichiometric matrix $N\in {\mathbb{Z}}^{n\;\times \;q}$, the reaction flux vector $v:{\mathbb{R}}_{\geq 0}^{n}\times {\mathbb{R}}_{\geq 0}^{p}\to {\mathbb{R}}_{\geq 0}^{q}$ and $k\in {\mathbb{R}}_{\geq 0}^{p}$ the parameter set.

## Methods

The brute-force method of determining the parameter region that satisfies a certain behavioral specification S⊆F usually proceeds by Monte Carlo sampling of parameter sets, generating corresponding trajectories according to (1), checking whether those satisfy *S *and finally retaining only those parameter sets that led to satisfied specification *S*. There are two immediate downsides of this approach. First, most draws will be unsuccessful for high dimensional parameter spaces, for tight specifications, or for both. Different approaches using an optimized sampling [11,17] have been developed to mitigate this problem, but are not solving it as they require convergence of the sampling. Second, drawing parameter points in ${\mathbb{R}}^{p}$ does not provide guarantees that those points belong to a connected domain of consistent parameter sets. Here we provide first attempts to tackle both problems.

The main idea is to locally linearize the forward map *F *around some point and then locally invert it. Hence, a small enough local patch in feature space can be mapped backward to a small patch in parameter space. By successively sampling expansion points in their neighborhoods (e.g. by the ball-walk algorithm [18]) we can systematically cover the entire specification *S *and obtain the corresponding parameter region. A series expansion of *F *around some initial parameter set *k*^0 ^reads

$$F\left(k^{0}+\text{d}k\right)=F\left(k^{0}\right)+{\left(\frac{\partial F\left(k\right)}{\partial k}\right|}_{k=k^{0}}\text{d}k+o\left(\text{d}k\right)$$

Defining d*f ≡ F *(*k*^0 ^+ d*k*) *− F *(*k*^0^) we see that a neighborhood d*f *in feature space to first order can be mapped backward using the Moore-Penrose pseudo-inverse

$$\text{d}k=L^{†}\text{d}f,$$

that we define with care as

(2) $$L^{†}\equiv \underset{\lambda \to 0}{\text{lim}}{\left(L^{T}L+\lambda I\right)}^{-1}L^{T}=\underset{\lambda \to 0}{\text{lim}}L{\left(LL^{T}+\lambda I\right)}^{-1},$$

where *L *denotes the linearized forward map and hence is just the *m × p *matrix

(3) $$L\equiv {\left(\frac{\partial F\left(k\right)}{\partial k}\right|}_{k=k^{0}}.$$

Note, that the limit in (2) exists even if the inverse of *L^T ^L *and *LL^T ^*do not exist. Such situations are encountered as soon as the number of specification features *m *are less than the number of parameters, i.e. the dimension *p *of the parameter space. Importantly, we can compute (3) efficiently using the variational equation for the system (1). Observe that

$$L=\frac{\partial }{\partial k}\psi \;o\;\varphi \left(k\right)={\left(\int_{0}^{T}\frac{\partial g\left(s,x\right)}{\partial x}\right|}_{x=x\left(s,k\right)}{\left(\frac{\partial x\left(s,k\right)}{\partial k}\right|}_{k=k^{0}}\text{d}s,$$

where the last terms in the integral is just the sensitivity of the solution of (1) to perturbations in *k *around *k*^0^. According to the variational equation the sensitivity obeys the following ordinary *n × p *matrix differential equation

(4) $$\frac{\text{d}}{\text{d}t}\frac{\partial x\left(t,k\right)}{\partial k}=N\left(\frac{\partial v\left(x,k\right)}{\partial x}\frac{\partial x\left(t,k\right)}{\partial k}+\frac{\partial v\left(x,k\right)}{\partial k}\right)\;\text{with}\;\frac{\partial x\left(0,k\right)}{\partial k}=0,$$

where we skipped the explicit dependency on *k*^0 ^for brevity. Note, that (4) is equivalent to the transient sensitivity analysis of metabolic networks [9,10], proposed as an extension of classical metabolic control analysis that only deals with steady state sensitivities. For a certain *k*^0 ^the sensitivity of the kernel *g *is a constant *m × n *matrix that can be computed explicitly. Thus, by jointly solving (1) and (4) for some *k*^0 ^together with

$$\frac{\text{d}}{\text{d}t}L\left(t\right)={\left(\frac{\partial g\left(s,x\right)}{\partial x}\right|}_{x=x\left(t,k^{0}\right)}{\left(\frac{\partial x\left(t,k\right)}{\partial k}\right|}_{k=k^{0}}\;\text{with}\;L\left(0\right)=0$$

up to time *T *we obtain the linearized map *L *= *L*(*T*). Hence, for every sampled *k*^0 ^and associated feature point *f *^0 ^we propose to design a feature ball

$$B_{f^{0}}\left(\delta \right)=\left\{f\in F|\;||f-f^{0}|{|}^{2}\leq \delta \right\}$$

and map it backward using *L*^†^. According to the singular value decomposition *L*^† ^= *U*Σ*V *with Σ a diagonal matrix with non-negative entries [16], the backward transformation needs to be a sequence of a rotation, a scaling and another rotation and hence the image of $B_{f^{0}}$ under *L*^† ^can only be a ellipsoid in the parameter space

$$\left\{L^{†}f|f\in B_{f^{0}}\left(\delta \right)\right\}\in {\mathbb{R}}^{p}.$$

Clearly, sampling a multivariate region with balls of same dimension allow for a complete coverage of the region - something that can only be extrapolated when using pointwise sampling [11]. The question to efficiently sample a region with balls has been addressed in computational geometry and efficient randomized algorithms are available [18].

We remark that the map *L *is not the best local approximation to *F*(*k*) in some norm sense. More specifically we can improve on *L *if we are giving additional samples of the neighborhood $B_{f^{0}}\left(\delta \right)$. Consider we draw another $k^{i}\in B_{k^{0}}$, then we can construct a rank-one update to *L*

(5) $${\tilde{L}}^{i}=L+\frac{\Delta F-L\Delta k}{||\Delta k|{|}^{2}}\Delta k^{T}$$

where Δ*F ≡ F*(*k^i^*) *− F*(*k*^0^) and Δ*k ≡ k^i ^− k*^0^. In particular, the rank-one term (5) captures the nonlinear part of *F*. From (5) it follows that the matrix ${\tilde{L}}^{i}$ satisfies the consistency property

(6) $${\tilde{L}}^{i}\left(k^{i}-k^{0}\right)=F\left(k^{i}\right)-f^{0}.$$

Thus, knowing how to construct rank-one updates over the domain of interest is equivalent to knowing *F*(*k*) locally. In fact, ${\tilde{L}}^{i}$ is the matrix closest to *L*, with respect to the Frobenius norm, that satisfies (6). Subsequently we will use this improved linear approximation to *F *to bound the error that one can incurrs if one uses the pseudoinverse *L*^† ^for the backward map. This will also provide means to determine the maximal ball size *δ *to stay below a certain error bound. We quantify the error in the feature space by the backward map followed by a forward map. That is, we want to find a *δ *such that

(7) $$||F\left(k^{0}+L^{†}\left(f-f^{0}\right)\right)-f|{|}_{2}\leq \varepsilon$$

for all $f\in B_{f^{0}}\left(\delta \right).$

Now suppose we know a bound *ρ*(*δ*) for the Frobenius norm of the rank-one perturbation, i.e. $||\tilde{L}-L|{|}_{F}\leq \rho \left(\delta \right)$ in the local domain of interest. Note, that *ρ*(*δ*) could and need to be estimated by sampling. Given a $f^{i}\in B_{f^{0}}\left(\delta \right)$ the maximal error of the inverse-forward map is

$$\underset{\tilde{L}:||\tilde{L}-L|{|}_{F}\leq \rho \left(\delta \right)}{\text{max}}||\tilde{L}L^{†}\left(f^{i}-f^{0}\right)-\left(f^{i}-f^{0}\right)|{|}_{2}$$

which is known from robust linear squares [16] to be equivalent to the error

$$||LL^{†}\left(f^{i}-f^{0}\right)-\left(f^{i}-f^{0}\right)|{|}_{2}+\rho \left(\delta \right)||L^{†}\left(f^{i}-f^{0}\right)|{|}_{2}.$$

Assuming that *L *has linearly independent rows, *LL*^† ^is the identity matrix and thereby the error simplifies to

$$\rho \left(\delta \right)||L^{†}\left(f^{i}-f^{0}\right)|{|}_{2}.$$

This result provides one way to determine the radius of the feature ball *δ *when relying on the pseudo-inverse

(8) $$\begin{array}{l} \underset{\delta }{\max\;\delta }\\ \text{subject}\;\text{to}\\ \rho \left(\delta \right)||L^{†}\left(f-f^{0}\right)|{|}_{2}\leq \varepsilon \\ ||f-f^{0}|{|}_{2}\leq \delta \end{array}$$

## Results

**Figure 2:**
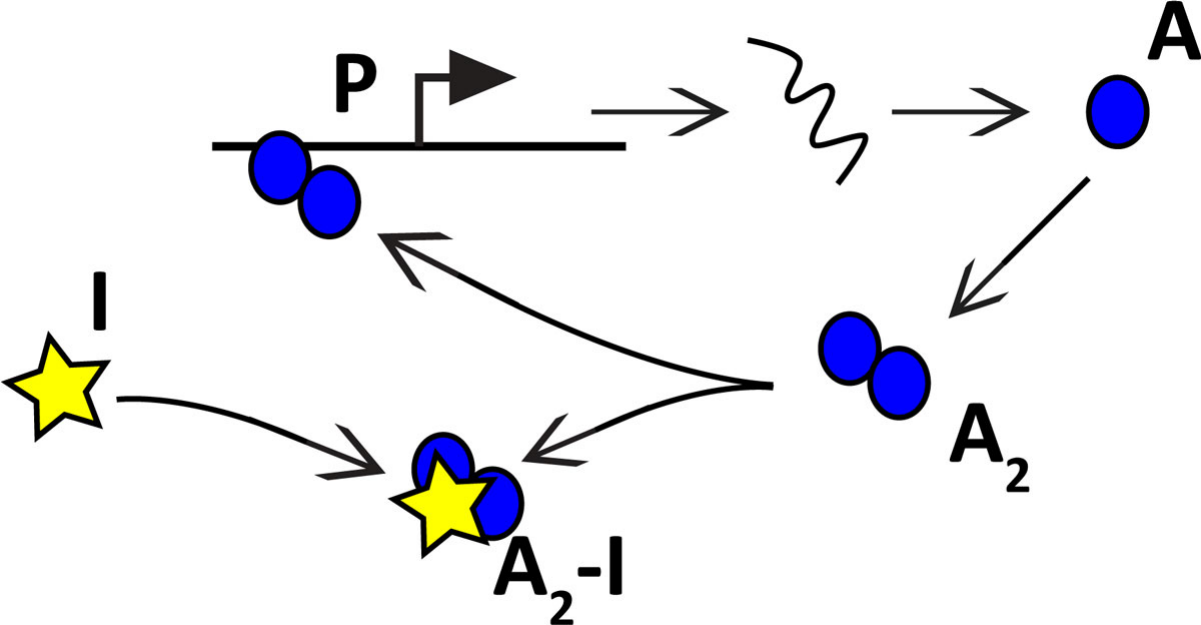
Simple transcriptional sensor construct. The dimerized form (*A*_2_) of a protein (*A*) is its own positive regulator; the inhibitor (*I*) tethers the dimer away in an inactive form (*A*_2 _*− I*).

As a proof of concept of our method, we applied it to a simple synthetic sensor construct [19]. The system is made of several gene copies (e.g. with plasmid transfection), expressing a protein that dimerizes and activates the gene by binding to the promoter. In presence of the inhibitor (input of the system), the dimer is trapped and cannot bind to the promoter. A schematic of the involved reactions is depicted in Figure 2.

The system is simulated according to mass-action and obeys

(9) $$\begin{array}{l} \frac{\text{d}x_{1}}{\text{d}t}=k_{1}(x_{5}^{0}-x_{5})+k_{2}x_{5}-k_{3}x_{1}\\ \frac{\text{d}x_{2}}{\text{d}t}=k_{4}x_{1}-2k_{5}x_{2}^{2}+2k_{6}x_{3}-k_{11}x_{2}\\ \frac{\text{d}x_{3}}{\text{d}t}=k_{5}x_{2}^{2}-k_{6}x_{3}-k_{7}x_{3}y(t)+k_{8}x_{4}-k_{9}(x_{5}^{0}-x_{5})x_{3}+k_{10}x_{5}-k_{11}x_{3}\\ \frac{\text{d}x_{4}}{\text{d}t}=k_{7}x_{3}y(t)-k_{8}x_{4}-k_{11}x_{4}\\ \frac{\text{d}x_{5}}{\text{d}t}=k_{9}(x_{5}^{0}-x_{5})x_{3}-k_{10}x_{5}-k_{11}x_{5}. \end{array}$$

where the states *x_i _*denote the concentration of mRNA, protein, protein-dimer and dimer-promoter complex, respectively. The quantities $x_{5}^{0}$ and *y*(*t*) refer the total number of promoters and the external inhibitor concentration, respectively. The nominal value and the meaning of the model parameters are summarized in Table 1. We remark that such continuous state-space model have their limitations for transcriptional circuits because they require several gene copies in order to neglect the discrete Boolean nature of a single gene.

**Table 1:** Nominal values and meaning of the kinetic parameters for the model of the synthetic sensor construct.

Basal transcription rate	*k*_1_	0.02 sec^−1^
Active-promoter transcription rate	*k*_2_	0.4 sec^−1^

mRNA degradation rate	*k*_3_	0.3 sec^−1^

Protein translation rate	*k*_4_	3 (nMsec) ^−1^

Dimerization rate	*k*_5_	0.1 (nMsec) ^−1^

Dimer dissociation rate	*k*_6_	0.001 sec^−1^

Inhibitor binding rate	*k*_7_	0.011 (nMsec)^−1^

Inhibitor unbinding rate	*k*_8_	0.2 sec^−1^

Dimer-promoter binding rate	*k*_9_	0.21 (nMsec)^−1^

Dimer-promoter unbinding rate	*k*_10_	0.2 sec^−1^

Protein degradation rate	*k*_11_	0.2 sec^−1^

Values are based on [19] and slightly adapted to obtain a desired threshold behavior.

For the specified behavioral features, we expect the dimer to drop quickly after introduction of inhibitor and then quickly regain a high level after the inhibitor is washed out of the medium. We also constrain the monomeric protein. The specification functionals are the integral of the absolute difference to some target value x* (*s*) for the monomer and the dimer concentration over two small time intervals for each. More specifically,

(10) $$\left[\begin{array}{c} \psi_{1}\left(x\right)\\ \psi_{2}\left(x\right) \end{array}\right]=\left[\begin{array}{c} \int_{0}^{T}w_{1}\left(s\right){\left[x_{2}\left(s,k\right)-x_{2}^{*}\left(s\right)\right]}^{2}\text{d}s\\ \int_{0}^{T}w_{2}\left(s\right){\left[x_{3}\left(s,k\right)-x_{3}^{*}\left(s\right)\right]}^{2}\text{d}s \end{array}\right]$$

where *w *is the temporal weight function chosen to be

(11) $$w_{i}\left(t\right)=\left\{\begin{array}{c} 1\;\text{for}\;t\in \left[t_{1},\;t_{2}\right]\cup \left[t_{3},\;t_{4}\right]\\ 0\;\text{otherwise} \end{array}\right)\;\text{for }i=1,2$$

**Figure 3:**
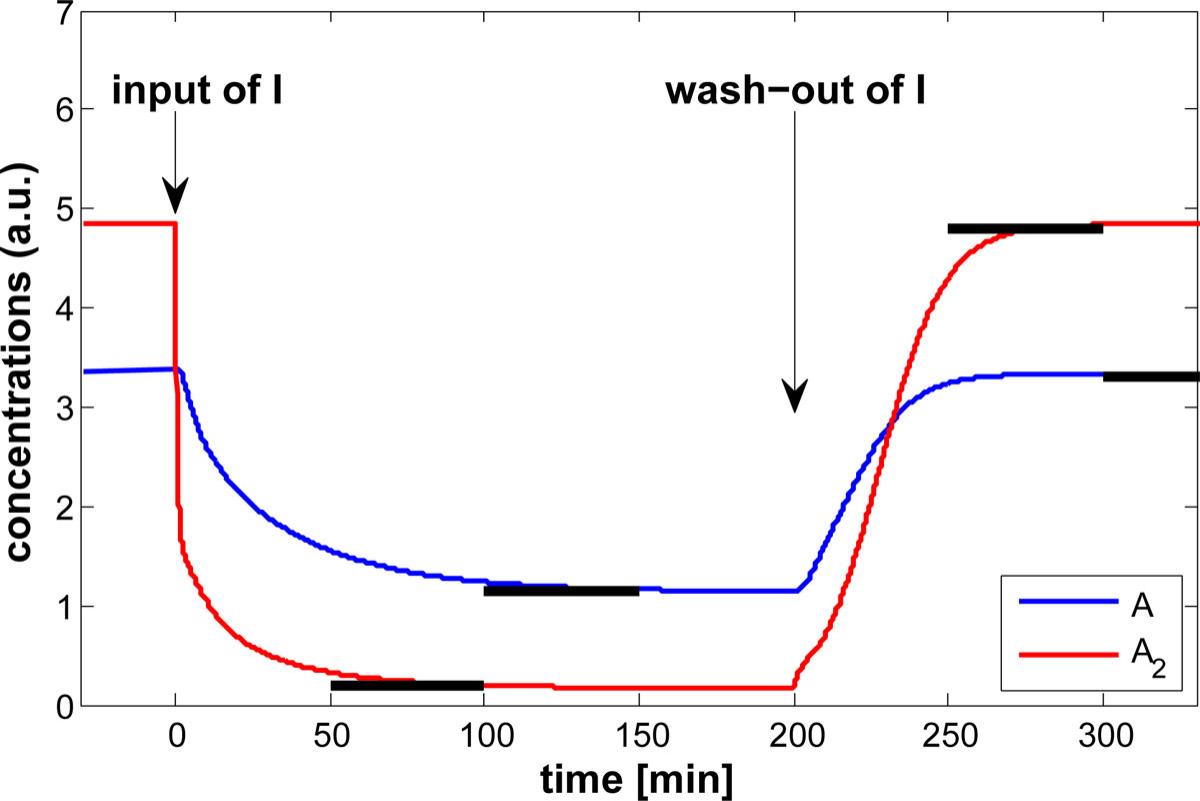
Time courses of monomer (*A*, *x*_2_) and dimer (*A*_2_, *x*_3_) concentration of (9) for an addition and removal of the inhibitor (*I*, *y*); the target values and time intervals chosen for the specification functionals are indicated by solid black lines.

The actual values for time-intervals for *w*_1_ and *w*_2_, as well as the target values are shown together with the trajectories for the nominal system (9) in Figure 3.

**Figure 4:**
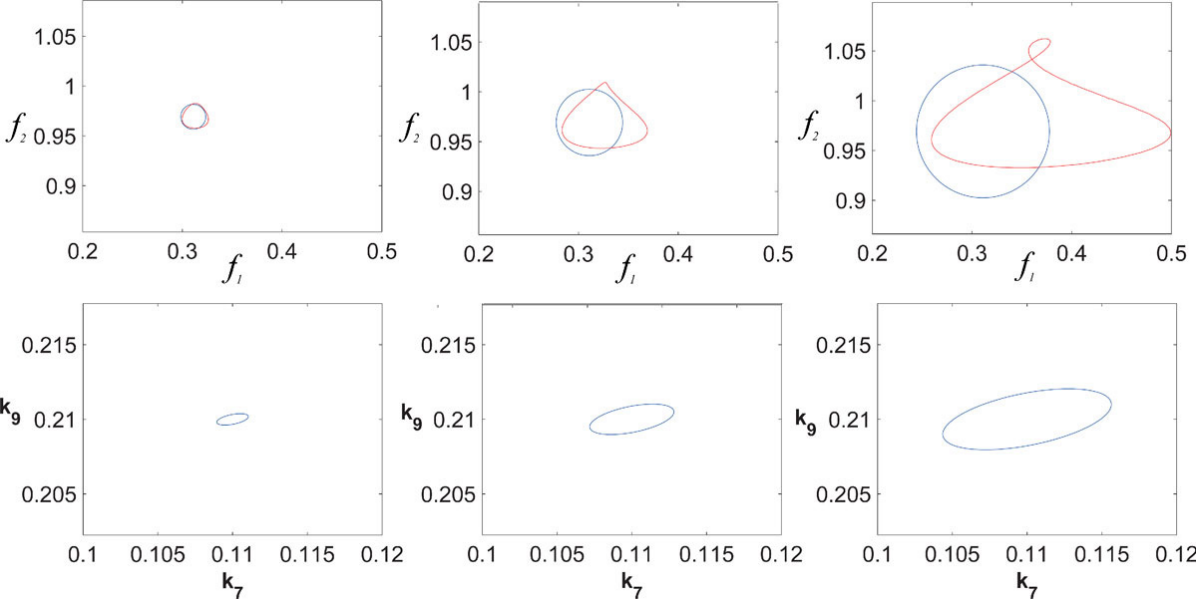
Contours of $B_{f^{0}}\left(\delta \right)$ (blue) in feature space (first row) are mapped back to the parameter space via *L*^† ^(second row) and mapped forward using *F *(red) for increasing size of *δ *(from left to right).

For this case study we assume that we have means to design the binding rate of the inhibitor to the dimer *k*_7_ and the binding rate of the dimer to the promoter *k*_9_. To assess the error incurred by the linearization we consider the reverse-forward mapping as described in (7). Hence for various size of *δ *we perform the inverse mapping with *L*^† ^and the forward mapping with *F*. If the inverse map is exact we should obviously obtain a ball with the same *δ*. Any deviation *ε *thereof reflects the approximation of *F^−^*1 by *L*^†^. In Figure 4 the images of $B_{f^{0}}\left(\delta \right)$ under *L*^† ^and *F ◦ L*^† ^are shown for various radii *δ*.

**Figure 5:**
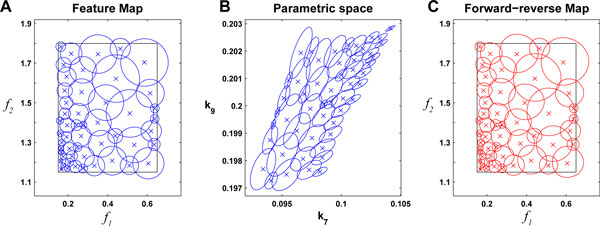
Covering a certain specification range *S *(black rectangle) by overlapping balls (A) which in turn yields overlapping ellipsoids in the parameter space (B). The precision of the mapping is illustrated by the reverse-forward map in (C). The centers of the balls are illustrated by crosses.

Hence, for an intermediate size of *δ *a good trade-off between approximation accuracy and sampling coverage is achievable. A systematic sampling of a predetermined specification area *S *would proceed by successively sampling overlapping balls with radii adapted to maintain *ε *under a certain value as illustrated in Figure 5. In this example, the coverage of the region *S *is above 98% using 50 balls of different radii. The lower left corner of the specification space (Figure 5A) maps to a strongly nonlinear region of the parameter space (upper right corner in Figure 5B) and therefore forces the use of smaller balls to keep the error in acceptable range. On the contrary, the upper right region of the specification space is more linear and larger balls can be used with limited relative error (Figure 5C).

## Conclusion

We presented a novel method to determine the parameter region of a biochemical reaction network that is consistent with a certain dynamical, behavioral specification. We defined specifications in a novel and general way that requires only the specification map to be once differentiable with respect to the states of the underlying differential equations. We showed that by locally linearizing this map we can solve the desired inverse problem of finding a parameter region for a given specification. As regions, instead of points, are mapped back to parameter space the scheme is in principle able to cover (given some regularity conditions) the feature and parameter space - something that is not possible with point-wise sampling. We also discuss means for estimating the size of the local neighborhood in order to guarantee certain approximation errors. The computational framework allows a very flexible definition of biologically relevant behavorial features and efficient determination of the corresponding parameter region. Hence, the range of experimentally modifiable parameters, such as promoter binding strength can be determined upfront before the experimental synthesis of a synthetic construct.

Throughout this work we only considered models based on ordinary differential equations, but the outlined framework can be extended to include stochastic dynamical models through the use of moment closure methods, for instance. In general, the specification functional will then involve the expectation operator and Monte Carlo sampling may be required to approximate it. Methods from stochastic sensitivity analysis [20] can be applied in order to perform the local inversion.

## Competing interests

The authors declare that they have no competing interests.

## Authors' contributions

HK, MH and JL devised the method, MH and JL implemented the algorithm and generated results for the case study. HK and MH wrote the paper.

## Declarations

Publication of this article was supported by the Swiss National Science Foundation (SNSF) grant number PP00P2_128503.

This article has been published as part of BMC Bioinformatics Volume 14 Supplement 10, 2013: Selected articles from the 10th International Workshop on Computational Systems Biology (WCSB) 2013: Bioinformatics. The full contents of the supplement are available online at http://www.biomedcentral.com/bmcbioinformatics/supplements/14/S10.
